# Threshold Effect of the Government Intervention in the Relationship Between Business Cycle and Population Health: Evidence From China

**DOI:** 10.3389/fpubh.2021.689870

**Published:** 2021-06-07

**Authors:** Kuang-Cheng Chai, Yang Yang, Zhen-Xin Cui, Yang-Lu Ou, Ke-Chiun Chang

**Affiliations:** ^1^Business School, Guilin University of Electronic Technology, Guilin, China; ^2^School of Economics and Management, Wuhan University, Wuhan, China

**Keywords:** government intervention, business cycle, population health, threshold effect, the Chinese government

## Abstract

China is an emerging country, and government intervention is always considered as an important part of the solutions when people facing challenges in China. Under the impact of the coronavirus disease 2019 (COVID-19) epidemic and the global economic downturn, the Chinese government quickly brought the epidemic under control and restored the positive economic growth through strong intervention. Based on the panel data of provincial level in China and the government intervention as the threshold variable, this paper empirically analyzed the non-linear effect of business cycle on population health by using the panel threshold regression model. The empirical results show that the impact of the business cycle on population health is significantly negative, and government intervention has a single threshold effect on the relationship between business cycle and population health. When the government intervention is below the threshold value, the business cycle has a significant negative effect on the improvement of the population health level; when the level of government intervention exceeds the threshold value, the relationship between business cycle and population health becomes significantly positive. To some extent, the conclusions of this paper can guide the formulation and revision of government health policy and help to adjust the direction and intensity of government intervention. The Chinese government and other governments of emerging countries should do more to harness the power of state intervention in their response to the business cycle.

## Introduction

The coronavirus disease 2019 (COVID-19) has become a worldwide epidemic, resulting in a serious health crisis due to the large number of infected persons and high death rate (1, 2), and population health is becoming a continuing focus of concern throughout the world. Health is an important component of human capital; some studies have advocated that health should be promoted to the core position of national productivity and become the central goal of economic growth (3). As the world's most populous country, the Chinese government plays an important role in preventing the spread of COVID-19 and promoting the level of population health. Through policy and institutional arrangement, government intervention can make up for market failure, eliminate poverty, and deal with public health emergencies. China's economic development has made great achievements, and along with the widening of rural medical insurance coverage, the government has done a lot of responsibility on these systems; these investments need to be backed by huge amounts of government funding. In this context, it is particularly important to study the effect of the business cycle on population health in China and find out the appropriate level of government intervention that should be maintained in responding to the business cycle. Governments especially in emerging countries need to take actions in fighting the epidemic and steadily improve population health throughout the business cycle.

We are on the cusp of a fourth industrial revolution, in which human-capable jobs are being replaced by automated algorithms, and robots are outperforming humans at complex tasks like robo advisers (4). Economic development is increasingly dependent on automation, more and more jobs are being replaced, and excessive life stress leads to a common state of subhealth. At the same time, people are living in a more affluent life; a large intake of high-protein, high-fat, and other foods leads to excess nutrition and insufficient exercise. What is more, people spend more time looking down to play with mobile phones, which is also bad for the health. Empirical studies have shown that government reductions in per capita health spending lead to higher mortality rates (5). Therefore, from the perspective of government intervention, the impact of business cycle on population health in China is worth studying. The World Health Organization believes that it is the political responsibility of governments to ensure that everyone has the right to healthcare. The instability, irregularity, and unpredictability of the medical and health demand led to the market failure and therefore the need for increased government intervention (6). On the one hand, the government influences the overall health level of population through public welfare policies; on the other hand, especially in some low-income areas, government financial expenditure has been an important financing channel for the effective implementation of the medical and health system.

Economic growth is affected by fluctuations in the price of oil (7). More attention should be paid to the impact of cyclically sensitive industries on people's health (8). For emerging countries that are highly dependent on energy or single industry, geopolitics greatly affects inflation and economic development, while the level of government intervention can help improve the immunity and efficiency against external shocks (9). The supply of basic health facilities and input of basic medical and nutrition guaranteed projects all need to be supported by the government's public budget. Governments can also understand the causes of oil price fluctuations to promote the health of energy markets (10). With the increasing availability of data, a growing number of scholars have confirmed the significant impact of government public health spending on health (11). Partisan conflicts in the United States affect the world economy and energy markets (12), and governments in emerging countries need to be more intrusive in shaping policy to respond appropriately to the business cycle and strive to improve population health.

This paper has the following contributions: first, based on China's provincial panel data, this paper empirically tests the impact business cycle on Chinese population health. Second, from the perspective of the degree of government intervention in China, this paper explores the non-linear characteristics of business cycle and population health by using the panel threshold regression model. Finally, based on the empirical results, this paper puts forward some suggestions on how to scientifically determine the level of government intervention in emerging country such as China and hope to provide experience and inspiration for other developing countries or underdeveloped countries to improve population health equity.

## Literature Review and Hypothesis

### Business Cycle and Population Health

Business cycle is the alternating or periodic fluctuation phenomenon of economic expansion and economic contraction, and the business cycle reflects how fast the economy is growing. With the rapid transformation of Chinese society, some people will appear impetuous, indifferent, and other negative psychology to a certain extent, which is considered to be very harmful to health. Research on the impact of the business cycle on health has been controversial. By studying the relationship between business cycle and mortality, some scholars have shown that economic growth will improve the health level of population (13). Economic growth will bring the increase in the overall income level of residents, and with the increase in income, the health expenditure will increase, and the health status will be improved (14). The economic downturn can cause a variety of health problems (15). While the studies of other scholars have shown that economic recession is conducive to the improvement of health: the mortality rate in Japan shows a trend of increasing during economic expansion and decreasing during economic recession (16); the fluctuation of the Spanish economy from 1980 to 1997 reflects that unemployment has a negative impact on mortality. When the economic growth and unemployment rate decrease, the mortality rate will increase (13). The health of adults (17) and newborns can be improved during economic recessions (18), a conclusion that has been similarly confirmed in several other countries (19, 20). Fluctuations in oil prices lead to wage arrears, which adversely affects people's incomes, and it is not good for people's quality of life (21). With the advance of urbanization in China, the fierce competition makes the employees more psychologically pressured; more and more people suffer from depression, which is not good for health. The industrial economic exhibition is behind the industrial wastes such as oil pollution, acid rain, and other issues, which has also brought new hidden dangers to population health.

Therefore, hypothesis 1 is proposed:

There is a significant negative correlation between business cycle and population health.

### Threshold Effects of Government Intervention

While factions in the United States were struggling fiercely, the Chinese government promoted better and faster development of the economy and people's livelihood through macro-control. The system with Chinese characteristics, which is characterized by the authority of political parties over the will of capital, has overcome the government failure of western countries. The high degree of government intervention is the internal root of China's success in defeating the epidemic (22). The influence of political factors on healthcare has received increasing attention in emerging countries. In a world where political partisanship is affecting the global economy, the government can also actively safeguard public safety and enhance people's confidence in the government (23). At present, China's resource allocation is not completely driven by the market but still relies on the administration-led mode of resource allocation management. In the face of increasing pressure to save energy and reduce emissions, the Chinese government has formulated various intervention policies in recent years, such as pollution total amount control, pollution costs, and environmental taxes. The Chinese government's energy conservation and emission reduction policy has improved environmental conditions (24) so as to contribute to population health. In fact, people's health depends on a variety of factors. For example, Labelle (25) believed that environment factor, mode of life and behavioral factor, biogenetic factor, healthcare services, and other influencing factors can influence population health. Studies have pointed out that the scale of government expenditure shows a non-linear growth trend along with economic growth, presenting an “inverted U-shaped” curve, and there is an optimal government size (26).

In addition to influencing population health through public health expenditure, the government also affects population health through environmental governance. The emission efficiency of pollution is affected by government intervention mechanism. Different stages of economic development have a significant impact on local governments. With rapid economical development, people's quality of life is rising. The public's environmental consciousness and health awareness will continually improve, and the desire for a high-quality environment will be even stronger. In this case, the diversified performance appraisal system with environmental protection as the core gradually replaced the local government performance appraisal system with gross domestic product (GDP) as the orientation. Local governments will be encouraged to strengthen environmental supervision and increase input in environmental protection (27); thus, it is conducive to population health. With the influence of the business cycle, the lack of government intervention may not be able to provide sufficient resources and help people to improve infrastructure and related healthcare level; especially in China, a major policy dominated by the government to carry out all health and poverty relief measures, more increased government intervention plays a role in the people's health level. In China, due to the special political system, a low level of political intervention may lead to the undoubted negative effects of the economic cycle on human health. However, if the government gives full play to the advantages of government intervention through environmental protection policies and medical care policies, it will be beneficial for the people to deal with the business cycle.Therefore, this paper puts forward the following hypothesis 2:

Government intervention has a threshold effect on the relationship between business cycle and population health.

## Data and Methodology

### The Data Source

Due to the lack of data in Xizang province, this paper uses panel data of 30 provinces in China during the decade of 2008–2017 to empirically test the relationship between business cycle, government intervention, and population health. Data are obtained from the official website of the National Bureau of Statistics of China and China National Statistical Yearbook.

### Variable Description

Referring to previous literature studies, this paper uses GDP growth rate (28, 29) to measure the business cycle as the explanatory variable. Using the continuous variable of annual GDP growth rate as the proxy variable of the business cycle can effectively avoid the possible errors caused by defining dummy variables to distinguish between the boom and recession periods. Perinatal mortality as a measure of population health is used as an explained variable in this paper (30). The threshold variable is local government intervention, which is measured by the proportion of local fiscal expenditure in regional GDP (31). The larger the ratio of local fiscal expenditure to regional GDP, the more significant the role of local governments in market resource allocation. One of the control variables in this paper is the urbanization rate, which is the proportion of urban population to total population (Ur), to measure the urbanization level of each province. In addition, there are also two types of demographic variables and environmental variables. Population characteristic variables include the aging degree of the population (Aging) and the education level of the population (Education), while environmental variables mainly include the road per capita (Road_per), the proportion of the secondary industry (S_indus), the average number of daily clients served per physician (Doc), and the proportion of the tertiary industry (T_indus). The definition of the variables can be seen in Table 1.

**Table 1:** Definition of variables.

**Variable**	**Measure unit**	**Definition**
GDPg	%	The GDP growth rate
Pmr	%	Perinatal mortality rate
Gi	%	Degree of government intervention
Doc	Average number	Average number of daily clients served per physician
Ur	%	Urbanization rate
Aging	%	Old-age dependency ratio
Education	%	Proportion of people who have at least 6 years of educational experience
Road_per	Square meters	Average square meters per person
S_indus	%	Proportion of secondary industry
T_indus	%	Proportion of tertiary industry

### Methodology

Based on the hypothesis mentioned above, the benchmark measurement model of fixed effects is first established:

(1) $$\begin{array}{l} Health_{it}=\alpha_{0}+\alpha_{1}GDPg_{it}+\alpha_{2}Doc_{it}+\alpha_{3}Ur_{it}+\alpha_{4}Aging_{it}\\ \;+\alpha_{5}Education_{it}+\alpha_{6}Road\_per_{it}+\alpha_{7}S\_indus_{it}\\ \;+\alpha_{8}T\_indus_{it}+\varepsilon_{it} \end{array}$$

where *i* represents the province; *t* represents the year; *Health*_*it*_ is measured by perinatal mortality rate (Pmr), which is the dependent variable; *GDPg*_*it*_ (measured by GDP growth rate of business cycle) is the independent variables; *Doc*_*it*_, *Ur*_*it*_, *Aging*_*it*_, *Education*_*it*_, *Road*_*per*_*it*_, *S*_*indus*_*it*_, and *T*_*indus*_*it*_ are the control variable; and ε_*it*_ is the random interference term.

However, according to the theoretical analysis above, the impact of the business cycle on population health does not necessarily show a simple linear relationship but may show a non-linear relationship with the different levels of government intervention. In order to further explore whether there is a relationship between business cycle and population health under different levels of government intervention, the panel threshold model was adopted in this study for empirical analysis.

(2) $$\begin{array}{l} Health_{it}=\omega_{i}+\gamma_{1}GDPg_{it}*I\left(Government\_inter_{it}\leq \sigma \right)\\ \;+\gamma_{2}GDPg_{it}*I(Government\_inter_{it}>\sigma )\\ \;+\gamma_{3}Controls+\varepsilon_{it} \end{array}$$

where ω_*i*_ is a constant term, *GDPg*_*it*_ is an independent variable, *Government*_*inter*_*it*_ is the threshold variable, σ is the threshold value, and ε_*it*_ is an error term to independent identical distribution.

## Empirical Results

### Descriptive Statistics

As can be seen from the Table 2, the standard deviation of GDP growth rate is 7.109%, and the difference between the maximum and minimum values is nearly 55%, indicating that the distribution of regional economic growth rate is relatively scattered, and there is a large gap in economic development. The standard deviation of aging and education level is small, indicating that the distribution of regional pension and education level in China is relatively concentrated. The standard deviation of urbanization rate is large, indicating that its distribution is relatively dispersed.

**Table 2:** Descriptive statistics.

**Variable**	**Obs**	**Mean**	**SD**	**Min**	**Max**
GDPg	300	12.308	7.109	−22.401	32.274
Pmr	300	6.689	2.836	2.020	19.170
Gi	300	23.334	9.854	8.744	62.686
Doc	300	6.902	2.497	3.130	15.200
Ur	300	54.722	13.207	29.110	89.600
Aging	300	13.067	2.681	7.440	20.597
Education	300	8.912	0.951	6.764	12.502
Road_per	300	14.275	4.461	4.040	25.820
S_indus	300	46.216	8.336	19.010	61.500
T_indus	300	43.365	9.362	28.600	80.560

**Figure 1:**
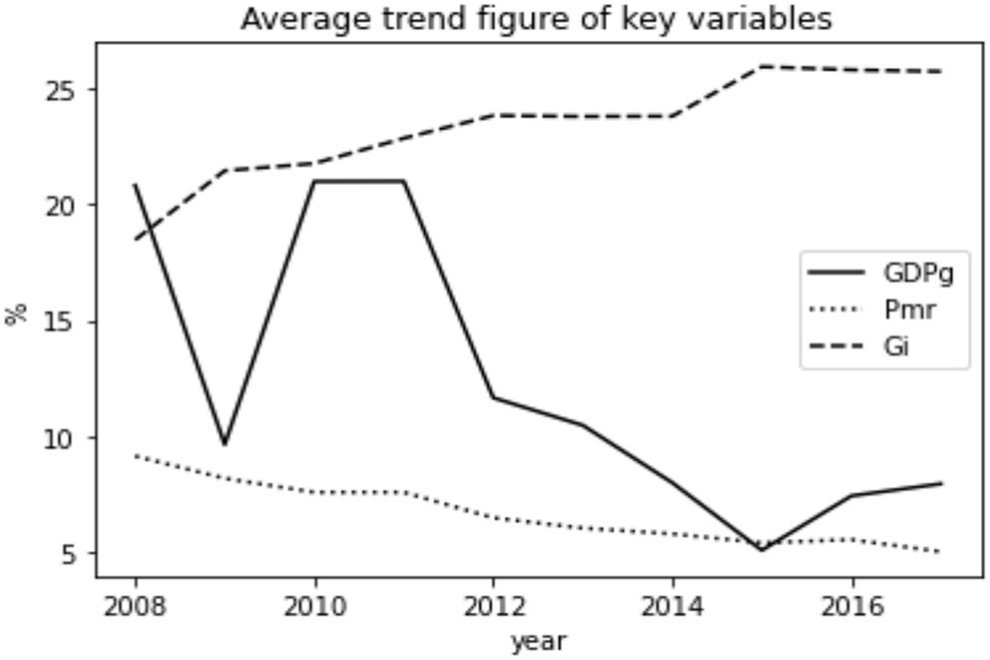
Average trend figure of key variables.

We can see clearly from the Figure 1 that the average number of average GDP growth rate of each province kept fluctuating between 10 and 20% during the period of 2008 and 2012 and hit the valley at around 5% in 2015, while that on government intervention showed an overall upward trend. At the same time, the perinatal mortality rates were slowly decreasing, indicating that the population health was getting better.

### Regression Results

As can be seen from the estimation results of fixed effect in Table 3, GDPg is negatively correlated with population health at the significance level of 10%. With the continuous development of economic conditions, the level of population health have also improved; thus, hypothesis 1 is verified. In order to ensure the robustness of the model, this paper selects GDP index as the alternative variable of the economic cycle. GDP index also shows significant cyclical characteristics and accurately describes the specific trend of the economy. The coefficient symbol and significance of GDP_index also prove the robustness of business cycle to population health. The coefficient symbols of other control variables are basically consistent with the existing studies.

**Table 3:** Regression result.

**Variables**	**Fixed effect**	**Fixed effect**
GDPg	0.020^*^	
	(1.73)	
GDP_index		1.139^***^
		(6.04)
Doc	0.195^**^	0.193^**^
	(2.17)	(2.14)
Ur	−0.115^***^	−0.118^***^
	(−3.93)	(−4.02)
Aging	−0.044	−0.044
	(−1.06)	(−1.08)
Education	−0.226	−0.204
	(−0.89)	(−0.80)
Road_per	−0.078^**^	−0.091^**^
	(−2.05)	(−2.45)
S_indus	0.102^**^	0.104^**^
	(2.48)	(2.55)
T_indus	0.160^***^	0.155^***^
	(3.48)	(3.35)
Constant	2.900	−121.264^***^
	(0.52)	(−4.82)
Year	Control	Control
Province	Control	Control
Observations	300	300
Adj *R*-squared	0.947	0.946

*** *p < 0.01,*

** *p < 0.05,*

* *p < 0.1*.

Next, the bootstrap method was used for 500 repeated sampling to determine the threshold value, and the validity of the threshold value was tested. The results are shown in Table 4.

**Table 4:** Threshold effect test.

**Model**	**Fstat**	***P*-value**	**Crit10**	**Crit5**	**Crit1**
Single threshold model	31.24^**^	0.038	23.979	29.153	41.726
Double threshold model	15.81	0.266	22.220	27.860	43.286

** *p < 0.05*.

It can be seen from Tables 4, 5 that when threshold effect is used for estimation, government intervention presents a single threshold effect on the impact of business cycle on population health, and the threshold value is 0.358. When the degree of government intervention is lower than 0.358, the coefficient of business cycle is highly significant and positively correlated with the perinatal mortality rate at the level of 1%. When the degree of government intervention is >0.358, the coefficient of business cycle is negatively significant at the level of 5%. It can be seen that government intervention has a threshold effect on the impact of business cycle on population health. When government intervention is lower than the threshold, the impact of business cycle on health becomes negatively correlated, and when government intervention exceeds the threshold, the impact of business cycle on population health becomes positively correlated. Therefore, the government should adjust the relationship between market level and strong government management, so as to release the promoting effect of economic growth on population health to the maximum extent.

**Table 5:** Threshold model result.

**Variables**	**Threshold regression**
GDPg_(it)_*I (<0.358)	0.039^***^
	(4.64)
GDPg_(it)_*I (≥0.358)	−0.044^**^
	(−2.55)
Doc	0.001
	(0.01)
Ur	−0.234^***^
	(−9.51)
Aging	0.553
	(0.15)
Education	−0.392^**^
	(−2.06)
Road_per	−0.123^***^
	(−3.10)
S_indus	0.046
	(1.08)
T_indus	0.064
	(1.42)
Constant	19.116^***^
	(5.59)
Observations	300
Adj *R*-squared	0.214

*** *p < 0.01,*

** *p < 0.05*.

## Conclusion and Suggestions

### Conclusion

This paper first uses a fixed-effect model to test the impact of business cycle on population health in China. In our study, the business cycle is defined as the GDP growth rate, and we find that the business cycle has a significantly negative impact on population health. In addition, according to the threshold effect test, it is found that government intervention has a single threshold effect on the relationship between business cycle and population health, with a threshold value of 0.385. In other words, when government intervention is lower than this level, business cycle will reduce the degree of population health, and when government intervention is above that level, the increase in economic growth will be rewarding and is good for population health. Strong government intervention in China is necessary to maintain the population health as the epidemic continues to spread around the world, which has led to great economic fluctuations. These conclusions provide valuable insights into the level of government intervention that policymakers should undertake in the face of an business cycle.

### Suggestions

The economic miracle that China has achieved in the past 40 years since reform and opening up has attracted the attention of the world, but how to properly handle the relationship between the government and the market is still a major issue that China and even the rest of the world should consider. According to the empirical results, in the economic downturn, the government needs to give full play to its function and formulate relevant development strategy to solve market failure caused by unreasonable resource allocation, thereby reducing the negative effect on the national health economic fluctuations. Governments in other emerging countries can also appropriately increase the degree of government intervention and provide policy support to their citizens in accordance with their national conditions.

Governments can also ensure that the mental and physical health of their people develop in harmony through cultural promotion and education. The authorities can improve people's satisfaction at work and get enough rest through education reforms and protecting the legal rights of workers. At the same time, it can carry out international experience exchange and cooperation to effectively solve the environmental pollution problems caused by rapid economic development and reduce the health hazards caused by ecological deterioration. In addition, the government can guide the society to raise funds for medical and healthcare and guarantee basic medical and health care and public health services. Big data can also be used to establish a prevention-oriented population health monitoring and evaluation platform, and a population health data sharing platform can be established through the Internet so as to improve people's own health awareness and optimize the health service system. Governments of all countries should join hands to support international cooperation in fighting the epidemic and promote the building of a health community for mankind.

## Data Availability Statement

The original contributions presented in the study are included in the article/Supplementary Material, further inquiries can be directed to the corresponding author/s.

## Author Contributions

K-CChai: conceptualization. K-CChan: methodology. YY, Z-XC, and Y-LO: formal analysis. K-CChai, YY, Z-XC, and Y-LO: investigation and validation. K-CChan and K-CChai: supervision. All authors contributed to the article and approved the submitted version.

## Conflict of Interest

The authors declare that the research was conducted in the absence of any commercial or financial relationships that could be construed as a potential conflict of interest.
